# Production of Dibromomethane and Changes in the Bacterial Community in Bromoform-Enriched Seawater

**DOI:** 10.1264/jsme2.ME18027

**Published:** 2019-02-15

**Authors:** Takafumi Kataoka, Atsushi Ooki, Daiki Nomura

**Affiliations:** 1 Faculty of Marine Science and Technology, Fukui Prefectural University 1–1 Gakuen-cho Obama, Fukui, 917–0003 Japan; 2 Faculty of Fisheries Sciences, Hokkaido University 3–1–1, Minato-cho, Hakodate, Hokkaido 041–8611 Japan

**Keywords:** dibromomethane (CH_2_Br_2_), dehalogenation, bacterial community, denaturing gradient gel electrophoresis, subarctic Pacific

## Abstract

The responses of bacterial communities to halocarbon were examined using a 28-d incubation of bromoform- and methanol-enriched subarctic surface seawater. Significant increases were observed in dibromomethane concentrations and bacterial 16S rRNA gene copy numbers in the treated substrates incubated for 13 d. The accumulated bacterial community was investigated by denaturing gradient gel electrophoresis and amplicon analyses. The dominant genotypes corresponded to the genera *Roseobacter*, *Lentibacter*, and *Amylibacter*; the family *Flavobacteriaceae*; and the phylum *Planctomycetes*, including methylotrophs of the genus *Methylophaga* and the family *Methylophilaceae*. Therefore, various phylotypes responded along with the dehalogenation processes in subarctic seawater.

Brominated, very short-lived halocarbons, such as bromoform (CHBr_3_) and dibromomethane (CH_2_Br_2_), with atmospheric lifetimes of 24 and 123 d, respectively (25), are potentially significant contributors to catalytic ozone loss in the troposphere and lower stratosphere (12). Although these two halocarbons are heterogeneously distributed along the depth of oceanic environments (26), their production, degradation, and transformation processes remain unknown. Abiotically, anoxic hydrolysis may result in the transformation of CHBr_3_ to CH_2_Br_2_ under limited conditions, such as with the activity of reduction catalysts or strong sunlight (1). The sources of these two halocarbons are considered to be associated with phytoplankton and macroalgae present in the subsurface of the ocean (20). Bacterial production and the loss of halocarbons have been proposed, with the dehalogenation of CHBr_3_ and subsequent production of CH_2_Br_2_ through microbial activity representing a potential mechanism (18). CH_2_Br_2_ concentrations correlate with the abundance of *Synechococcus*, picoeukaryotes, and heterotrophic bacteria as well as with the total chlorophyll *a* concentration in the Atlantic Ocean (11). These findings suggest that CH_2_Br_2_ production is attributable to biogeochemical processes related to the phytoplankton biomass, such as degradation by heterotrophic bacteria and grazing by animals. To date, only three strains of marine class *Alphaproteobacteria* (*Phaeobacter gallaeciensis*, *Roseobacter denitrificans*, and *Rhodobacter vinaykumarii*) are known to decompose CHBr_3_ to CH_2_Br_2_ (6). The bacterial production of CH_2_Br_2_ occurs from a relatively early exponential phase of bacterial growth. In contrast to CHBr_3_, CH_2_Br_2_ has not been shown to be decomposed by these three strains. Thus, aquatic bacteria may act as new sinks for CHBr_3_ and new sources for CH_2_Br_2_ in marine environments. The purposes of the present study were to evaluate the responses of bacterial communities to the addition of CHBr_3_ to subarctic surface seawater and to identify the bacteria phylotypes involved in CH_2_Br_2_ production as a response to this addition using a 28-d incubation.

Seawater samples were collected from the eastern coast of Hokkaido, the northern area of Japan, located in the subarctic gyre of the western North Pacific (42.3°N, 140.6°E) by T/V *Ushio-Maru* on December 12^th^, 2016. Seawater was taken from a depth of approximately 5 m, at which temperature and salinity were +8.4°C and 33.9, respectively, and then filtered through a Nuclepore filter with a pore size of 10 μm (47 mm in diameter; Whatman) to remove large-sized zooplankton and phytoplankton. Four 1.2-L amber glass bottles were filled with filtered water and closed using a Teflon-coated inter-lid (GL45 and A17200-001, respectively; Shibata Scientific Technology) without head space to avoid gas exchange between the inside and outside of the bottle. Glass bottles were stored in a refrigerator (+4°C) onboard until further processing. To imitate the natural environment in which the CHBr_3_ concentration, which reached 1.2 nmol L^−1^, matched the phytoplankton biomass distribution (17), 8 nmol of CHBr_3_ (027-10142; Wako Pure Chemical Industries) was diluted in 99 μmol of methanol (CH_3_OH) (139-08821; Wako Pure Chemical Industries) and added to the glass bottle (labeled as “treatment”) in the laboratory on land within 24 h of sampling. Since it was not possible to predict the production rate of CH_2_Br_2_ from CHBr_3_, a sufficient amount of CHBr_3_ (8 nmol) was added to the treatment samples (a final concentration of 6.67 nmol L^−1^). Methanol was used as a solvent because CHBr_3_ is hardly soluble in water and is produced by phytoplankton in natural seawater (5). A bottle sample without the added reagents was also prepared (labeled as the “control”). No abiotic and only CH_3_OH controls were also prepared to save water and incubation space. Duplicate measurements for organic gas concentrations and bacterial parameters were conducted for each sample.

In the seawater incubation, glass bottles were stored at 4°C for 28 d in the dark to avoid photodegradation of the substrates (CHBr_3_ and CH_2_Br_2_) (1) and the growth of phytoplankton. In the analysis of organic gas concentrations, 30 mL of a subsample was carefully transferred into a 30-mL amber glass vial (Nichiden-Rika Glass) on days 0, 13, and 28. A mercuric chloride solution (saturated HgCl_2_; 200 μL) was added to the glass vials to stop biological activity. Vials were sealed with a Teflon-lined septum and aluminum cap (Nichiden-Rika Glass) and stored in the refrigerator (+4°C) until further analyses. In analyses of bacterial abundance and composition, a 200-mL subsample was filtered (pore size of 0.2 μm and diameter of 47 mm) and rapidly frozen at −30°C. CH_2_Br_2_ and CHBr_3_ concentrations were assessed using purge-and-trap and gas chromatography mass spectrometry (GC-MS) methods (16) (details in supporting information).

In molecular analyses, genomic DNA was extracted from the whole filter using a FastDNA Spin kit (Nippon gene) and quantified using a Qubit 3.0 fluorometer (Thermo Fisher Scientific). Real-time quantitative PCR (qPCR) was performed to quantify bacterial 16S rRNA gene copy numbers using the primer set of 1369F and 1492R (Table S1) as previously described (9) with some slight modifications (supporting information). Bacterial communities were investigated by denaturing gradient gel electrophoresis (DGGE) using the primer set of 907R and 341F attaching a GC-clamp to the 5′-end of the forward primer under conditions (shown in Table S1) described previously (7). To semi-quantitatively compare the genotype composition of the 16S rRNA gene, amplicon sequencing of the primer set of 341F and 805R was conducted (supporting information). The sequence obtained from the DGGE band and representative sequences of operational taxonomic units (OTU) from the amplicon sequencing analysis were compared with the GenBank database using BLASTn, and aligned against the SILVA alignment (SSU Ref NR 128) to obtain a NJ tree.

The time evolution of CH_2_Br_2_ concentrations and bacterial abundance, assessed by 16S rRNA gene copy numbers during the incubation, is shown in Fig. 1. CH_2_Br_2_ concentrations increased with time from 10.8 to 25.1 pmol L^−1^ in the treatment samples, but remained constant in the control samples (4.0±0.1 pmol L^−1^) (mean±standard deviation) (Fig. 1A). The difference observed in CH_2_Br_2_ concentrations between the control and treatment bottles on day 0 was presumably due to contamination of the CHBr_3_ solution (0.1% of CHBr_3_ solution). Bacterial abundance was greater in the treatment bottles than in the control bottles and showed a marked increase from 9.7×10^3^ and 1.34×10^4^ copies mL^−1^ on day 0 to 4.6×10^4^ and 1.9×10^4^ copies mL^−1^, respectively, on day 13 (Fig. 1B). The low copy number on day 28 indicates the death phase of the batch culture caused by a nutrient shortage after increases in some genotypes. CHBr_3_ consumption was not clearly detected (Fig. S1) because the initial concentration of this halocarbon was so high that its degradation into CH_2_Br_2_ was smaller than the variance due to handling errors. Although the utilization of CHBr_3_ and/or CH_3_OH, which was added as a solvent, was not measurable, the present results suggest that the sea surface bacterial community in the eastern coast of Hokkaido is involved in the production of CH_2_Br_2_.

A DGGE analysis was conducted to monitor the bacterial community during the experiment (Fig. 2). The DGGE band pattern in the control bottles showed a small change in the band position during the incubation period, with some bands showing marked changes in their intensities. Conversely, some intense DGGE bands in the treatment bottles specifically appeared on day 13 (bands of vg-1, -2, and -3 in Fig. 2) along with the disappearance of most of the DGGE bands that were present on day 0. A 16S rRNA gene amplicon sequencing analysis was conducted on day 13 to compare genotype richness between the control and treatment samples. The number of OTUs, which were grouped based on a similarity level of 97%, was lower in the treatment bottles than in the control bottles (Fig. S2), indicating that the bacteria community was enriched with particular genotypes that adapted to amended CHBr_3_ and/or CH_3_OH. To examine the accumulated OTUs, the ratio of the relative abundance of each OTU in the treatment bottles to that in the control bottles was calculated by the average of the replicates on day 13 (Table S2). Among the 960–961 OTUs in the treatment bottles, 23 showed >10-fold higher values for this ratio than OTUs in the control bottles or were exclusively detected in the treatment bottles with a relative abundance >0.01%. One OTU of VSN07, which was close to the genus *Methylophaga* of class *Gammaproteobacteria* (Fig. 3), was significantly dominant in the treatment bottles (65.9 and 73.5%). *Methylophaga*-related genotypes were also previously shown to increase their abundance in methyl bromide-amended seawater during phytoplankton blooms in the English Channel (15). Thus, the present results suggest that *Methylophaga*-related genotypes respond to CHBr_3_ and/or CH_3_OH in the eastern coast of Hokkaido.

Sequence analyses revealed the phylogenetic positions of the genotypes that responded to the addition of CHBr_3_ and CH_3_OH after an incubation for 13 d (Fig. 3). Four sequences from the DGGE analysis were close to those obtained from phytoplankton blooms or were related to methanotrophic strains (8). Two sequences of vg-3 and vg-4 belonged to the family *Rhodobacteraceae* in the class *Alphaproteobacteria*. The vg-3 genotype, which was close to the recently defined taxa of *Amylibacter manrinus* (22), belongs to the NAC11-7 clade comprising various genotypes detected after phytoplankton blooms (2, 21, 24). The vg-4 genotype was close to *Lentibacter algarum* isolates obtained from massive algal blooms in China (10). The sequence of vg-1, which belongs to the family *Flavobacteriaceae*, is close to that of an abundant genotype in phytoplankton blooms after iron fertilization (clone C114Chl356 [19]). Thus, the major genotypes responding to CHBr_3_ and/or CH_3_OH were related to natural phytoplankton blooms. The results of the amplicon sequencing analysis corresponded to those of the DGGE analysis, except for the vg-3 genotype to which no close sequences were detected (Fig. 3 and S3). Most of the responding genotypes in the treatment bottles (Table S2) were similar to those identified from the DGGE sequences (*i.e*., vg-1, -2, and -4). The family *Methylophilaceae* in the class *Betaproteobacteria* is a major phylogenetic group of the OM43 clade (4, 13), which possesses the methanol dehydrogenase enzyme (XoxF) (3). Thus, the genotypes of VSN04–06 may have been involved in methanol (CH_3_OH) degradation even if their relative abundance was low (Table S2). The other methylotroph-related genotypes (VSN07–14 and 21) were close to the genus *Methylophaga* in the class *Gammaproteobacteria*, which uses CH_3_OH as a source of carbon (23). The diverse and abundant genotypes were only close to uncultured bacteria-derived sequences. Neufeld *et al*. (14, 15) also found diverse uncultured CH_3_Br-assimilating genotypes of the genus *Methylophaga* (SIP MBr clade); however, their sequences were different from those obtained in the present study. Although it currently remains unclear whether the methylotrophs detected in the present study contribute to the production of CH_2_Br_2_, the diverse but local genotypes that formed a clade in the phylogenetic tree (Fig. 3) increased their abundance in response to the addition of CHBr_3_ and/or CH_3_OH. Conversely, one genotype of VSN03, belonging to the class *Alphaproteobacteria*, was close to *P. gallaeciensis*, which produces CH_2_Br_2_ in CHBr_3_-amended media during the early growth phase (6), suggesting that VSN03 was a source of CH_2_Br_2_ in our experiment. The other abundant genotypes in the treatment bottles belonged to the genus *Lentibacter* in the class *Alphaproteobacteria* and the phyla *Bacteroidetes* and *Planctomycetes*. Although their capacity to metabolize halogenated alkanes remains unknown, there is scope for assessing their metabolism because CFB and some unknown phylotypes have been shown to incorporate ^13^CHBr_3_ (15). A second diverse clade formed by VSN01, 02, and 20 within the genus *Lentibacter* may also metabolize C1 methyl halides.

In conclusion, the present results suggest that the eastern coast of Hokkaido is inhabited by a bacterial community that responds to CHBr_3_ and/or CH_3_OH and plays a major role in dehalogenation. Pure cultures of these genotypes will provide insights into the role of halocarbon metabolism in carbon and halocarbon cycles in the ocean.

## Supplementary Information



## Figures and Tables

**Fig. 1:**
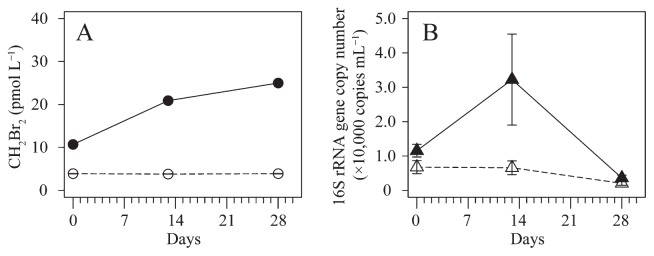
Time evolution of (A) CH_2_Br_2_ concentrations and (B) bacterial abundance assessed by 16S rRNA gene copy numbers during the incubation experiment. The closed symbol indicates the treatment and the open symbol indicates the control. Error bars indicate variance in duplicate bottles.

**Fig. 2:**
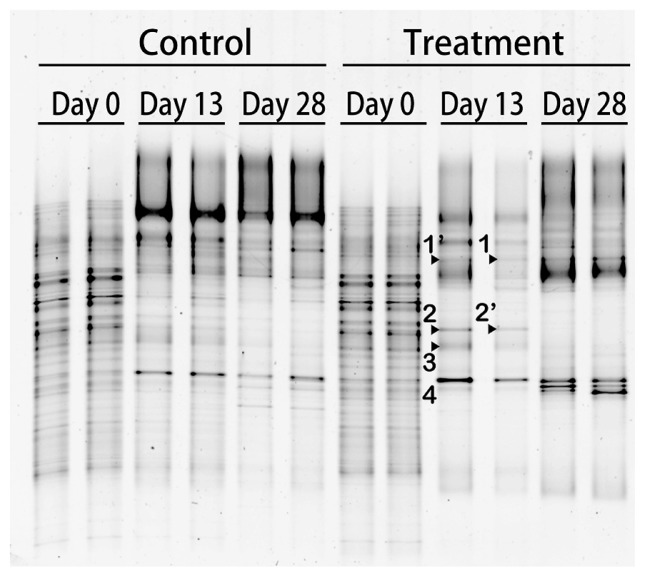
DGGE profiles of 16S rRNA gene fragments from incubation experiments in duplicate bottles. Treatment and control indicate an incubation with bromoform (CHBr_3_) and methanol (CH_3_OH) and without additives, respectively. The day on the top of each lane indicates the elapsed day after the initiation of the treatment (day 0). The nucleotide sequences of the labeled bands were elucidated and corresponded with sequence names in Fig. 3. The apostrophe indicates similar sequences.

**Fig. 3:**
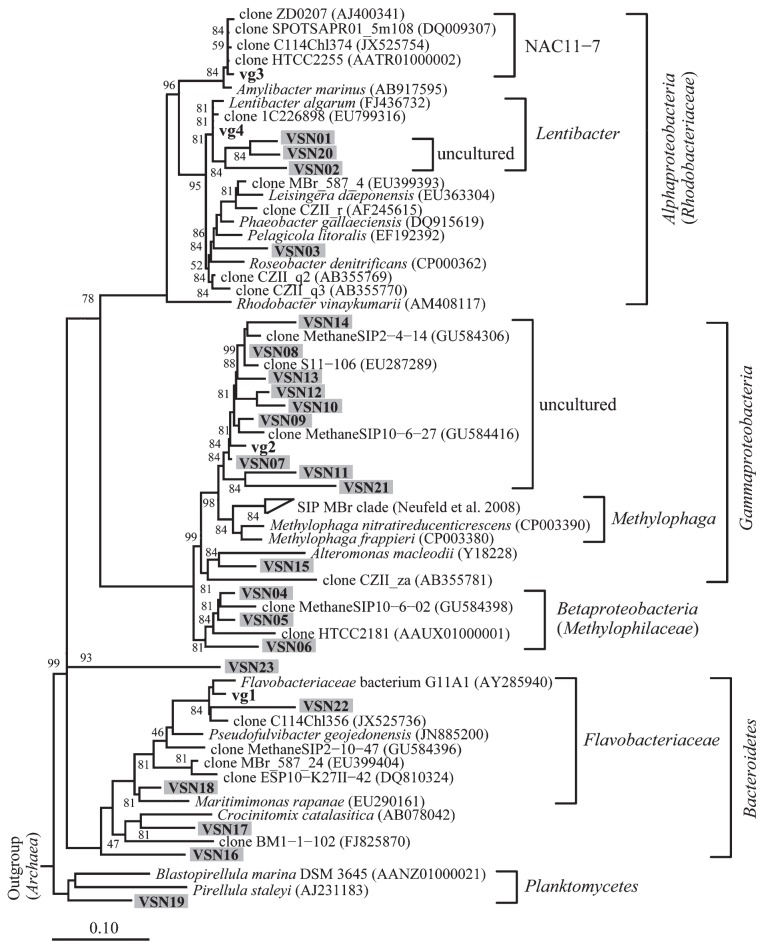
A neighbor-joining tree of partial 16S rRNA gene sequences of four cut DGGE bands (*ca*. 550 bp) that correspond to bands in Fig. 2 (bold font) and 23 OTUs from 97% similarity-based OTUs (*ca*. 427 bp) that were more abundant in the treatment (shown in Table S2 [shaded font]). Bootstrap values (per 50000 resampling) >45% are shown. A prefix of query sequence, “vg”, indicates DGGE bands from VOC-enriched treatment and “VSN” indicates sequences obtained from next-generation sequencing (NGS) that increased in VOC-enriched treatments. The scale bar indicates the substitution rate.
