# Supplementary material for: Production of Dibromomethane and Changes in the Bacterial Community in Bromoform-Enriched Seawater
Source: Microbes Environ. 2019 Feb 15;34(2):215–8. doi: 10.1264/jsme2.ME18027 (PMC6594743; doi:10.1264/jsme2.ME18027)
Supplement: Supplementary file 1 [file 34_215_s1.pdf]

Supporting text

Supporting Materials and Methods.

Detail methods for CH_2Br_2 and $CHBr_3$ analysis.

The dibromomethane (CH_2Br_2) and the bromoform ($CHBr_3$) were determined by a purge-and-trap (P&T) and gas chromatography-mass spectrometry (GC-MS) method (7). The total volume of seawater in the sample bottle was transferred to a custom-made bubbling vessel at +58°C by nitrogen carrier at 20 mL min^{-1}. The CH_2Br_2 and $CHBr_3$ in the water were purged with the nitrogen carrier at 65 mL min^{-1} for 30 min, and simultaneously trapped with TenaxTA (10 mg) resin in a stainless tube (1/16' diameter) at –90°C. The trapped CH_2Br_2 and $CHBr_3$ were transferred with nitrogen carrier at +200°C to a pre-concentration GC-MS system (Agilent 7890A, 5975C; Agilent Technologies, Santa Clara, CA, USA) (7). A gravimetrically prepared standard gas (Taiyo Nissan, Inc., Tokyo) containing CFC-11 HCFC-22, CH_2Br_2 and $CHBr_3$ at initial concentrations of 10 ppb (dry air mole fraction) in 2012 was analyzed with the pre-concentration–GC-MS system in 2017. Daily analysis of standard gas including CFC-11 and HCFC-22, which are stable in the standard gas cylinder, was used for checking conditions of pre-concentration/GC-MS system. To calibrate for the concentrations of CH_2Br_2 and $CHBr_3$ in the standard gas, we prepared a methanol-based liquid standard containing these compounds. The methanol-based standard solution was diluted in pure water just before the Purge & Trap procedure. The standard samples (standard gas and methanol based diluted standard) were measured by the same GC-MS system. The concentrations of CH_2Br_2 and $CHBr_3$ in the gravimetrically prepared standard gas in 2017 were 6.7 ppb and 2.1 ppb, respectively. We used these concentrations for calibrations. A diluted standard solution was introduced to the P&T GC-MS system to calibrate for CH_2Br_2 and $CHBr_3$ in seawater samples. The precision of standard solution measurements was ±2% (n=6).

Detail methods for quantitative real-time PCR.

For A 20-μL of qPCR reaction mixture was prepared with containing a 1 μL portion of ten times diluted genomic DNA (gDNA), 0.25 μM of each primer, and a 1× QuantiTect SYBR Green PCR Master Mix (QuantiTect SYBR Green PCR Kit, Qiagen). The cycling condition was as follows: after heating +95 ºC for 15 min to activate the hot start *Taq* polymerase, 40 cycles of +94 ºC for 30 sec, annealing at +55 ºC for 30 sec, and elongation at +72 ºC for 30 sec. For quantitative enumeration, standard curves were constructed from ten-fold serial dilutions between 1.0×10^1 and 1.0×10^8 copies of the PCR amplicon generated from M13 primer set from a plasmid (pCR®2.1, Invitrogen) containing target 16S rRNA gene. Non-specific amplification was determined by melting curve analysis followed by the qPCR cycles.

Amplicon sequencing and data analysis of 16S rRNA gene.

Amplicon library for high throughput sequencing analysis of bacterial 16S rRNA gene was constructed using a set of fusion primer for Illumina sequencing (Table S2). PCR amplification was

performed in 25 μL reaction mixture containing 1× KAPA HiFi HotStart ReadyMix (KAPA Biosystems), 0.3 μM of each primer and 3 μL of ten times diluted gDNA that correspond to 0.4–1.3 ng of gDNA under cycling condition as following: after heating 94 ºC for 15 min to activate the hot start DNA polymerase, 30 cycles of 94 ºC for 30 sec, annealing at 55 ºC for 30 sec, and elongation at 72 ºC for 30 s. Amplicon was purified and labeled with index primer set attaching to the both 5' and 3' end (NEBNext Multiplex Oligos, New England BioLabs), then sequenced using MiSeq Reagent kit v2 for 2 × 250 bp chemistry (Illumina). All of the generated sequence reads were de-multiplexed according to the index primers and processed with the software package Claident ver. 0.2.2017.07.26 (13) as descried previously with a minor modification (3). Briefly, the sequence reads were quality controlled by filters that removed reads containing any single mismatch and ambiguous bases in the index tag or primer sequences, and remained paired-end reads were combined with >50 bp overlapping end of each read by VSEARCH (10). The combined reads that have >300 bp length was quality controlled by a filter that remove reads containing a quality value of less than 30. Then potentially chimeric sequences were removed by the VSERCH. Next remaining reads were used to establish operational taxonomic units (OTUs) using 97% cut-off level. A representative sequence of each OTUs was filtered to pickup the ribosomal RNA (rRNA) gene sequences using riboPicker (11) with referring to SILVA database (SSURefNR 128; (9)). After removed the singleton throughout analyzed libraries, sequences belonged to archaea (13.5 ± 8.3 reads/sample; 3.3 ± 1.5 OTUs/sample, in 4 libraries) were removed to determine the compositional change of major bacterial taxa. The remaining reads of each library were converted to the relative read number per 100,000 reads. For phylogenetic analysis, represent sequences of OTUs from amplicon analysis and of DGGE bands were aligned against the SILVA alignment (SSU Ref NR 128; (9)) using SINA program (8). For phylogenetic analysis, a reference NJ tree was constructed using sequences close to those obtained with the SILVA database and BLAST search. Then obtained sequences were inserted without changed the tree topology using ARB software (4).

Statistical analysis.

Rarefaction curves were calculated using vegan package ver. 2.4 (6). Heatmap was calculated based on the Bray-Curtis dissimilarity distance of class-level OTU composition using gplots package ver. 3.0.1. All statistic analyses were conducted using software R ver. 3.3.2 (http://cran.r-project.org).

Supporting Table.

Table S1. Primer sequences and the conditions of PCR for DGGE, DGGE gel and qPCR for 16S rRNA gene.

					PCR	DGGE		
Application	Estimated length (bp)	Primer name	Primer sequences [5' to 3']	AT (ºC)	Cycle no. (cycles)	AG Conc. (%)	Denaturing range of AG (%)	Reference
DGGE	550 bp	341F<br>907R*	CCTACGGGAGGCAGCAG<br>CCGTCAATTCMTTTGAGTTT	65–55†	35	8	25–70	(5)
qPCR	142 bp	Bact-1369F<br>Prok-1492R	CGGTGAATACGTTCYCGG<br>GGWTACCTTGTTACGACTT	55	40	–	–	(12)
Illumina sequencing	16S rRNA (323 bp)	341F**<br>805R**	CCTACGGGNGGCWGCAG<br>GACTACHVGGGTATCTAATCC	55	30	–	–	(5)<br>(2)

* attached GC clump sequence: 5'-CGCCCGCCGCGCCCCGCGCCCGTCCCGCCGCCCCCGCCCG-3' [to 5' end of R primer]

** attached NEBNext adapter for Illumina with a bridge sequence of 0–5 N nucleotide (1)

† Touch down protocol –0.5ºC at first 20 cycles was applied to PCR

AT indicates annealing temperature; AG indicates acrylamide gel

Table S2. Relative read abundance (i.e., read number normalized to 100,000 reads in each bottle), and ratio of the averaged value in the treatment bottles to that in the control bottles. OTUs showing >10-fold higher abundance in the treatment bottles than in the control or an abundance > 0.01% (10/100,000 reads) in the treatment bottles but below the detection level in the control (which is distinguished by the prefix "+") were listed.

			Relative read abundance (per 100000 reads)				
SILVA taxonomic path	Sequence name[a]	Accession number	Control-1	Control-2	Treatment-1	Treatment-2	Ratio between Control and Treatment[b]
Bacteria;*Proteobacteria*;*Alphaproteobacteria*;*Rhodobacterales*;*Rhodobacteraceae*;*Lentibacter*;	VSN01	LC387491	0.0	1.4	7.1	14.5	15.6
Bacteria;*Proteobacteria*;*Alphaproteobacteria*;*Rhodobacterales*;*Rhodobacteraceae*;*Lentibacter*;	VSN02	LC387492	0.0	1.4	24.8	11.9	26.5
Bacteria;*Proteobacteria*;*Alphaproteobacteria*;*Rhodobacterales*;*Rhodobacteraceae*;	VSN03	LC387493	0.0	1.4	19.5	7.9	19.8
Bacteria;*Proteobacteria*;*Betaproteobacteria*;*Methylophilaceae*;*Methylotenera*;	VSN04	LC387494	2.9	8.3	40.8	88.4	11.6
Bacteria;*Proteobacteria*;*Betaproteobacteria*;*Methylophilaceae*;*Methylotenera*;	VSN05	LC387495	8.6	4.2	216.3	200.6	32.7
Bacteria;*Proteobacteria*;*Betaproteobacteria*;*Methylophilaceae*;	VSN06	LC387496	0.0	1.4	19.5	18.5	27.4
Bacteria;*Proteobacteria*;*Gammaproteobacteria*;*Nitrosococcales*;*Methylophagaceae*;	VSN07	LC387497	5945.4	7328.6	65905.5	73538.9	10.5
Bacteria;*Proteobacteria*;*Gammaproteobacteria*;*Nitrosococcales*;*Methylophagaceae*;	VSN08	LC387498	2.9	5.5	152.5	372.1	62.4
Bacteria;*Proteobacteria*;*Gammaproteobacteria*;*Nitrosococcales*;*Methylophagaceae*;	VSN09	LC387499	7.2	5.5	147.2	208.5	28.0
Bacteria;*Proteobacteria*;*Gammaproteobacteria*;*Nitrosococcales*;*Methylophagaceae*;	VSN10	LC387500	0.0	1.4	10.6	25.1	25.8
Bacteria;*Proteobacteria*;*Gammaproteobacteria*;*Nitrosococcales*;*Methylophagaceae*;	VSN11	LC387501	0.0	0.0	8.9	11.9	+10.4
Bacteria;*Proteobacteria*;*Gammaproteobacteria*;*Nitrosococcales*;*Methylophagaceae*;	VSN12	LC387502	0.0	0.0	47.9	51.5	+49.7
Bacteria;*Proteobacteria*;*Gammaproteobacteria*;	VSN13	LC387503	0.0	0.0	56.7	1.3	+29.0
Bacteria;*Proteobacteria*;*Gammaproteobacteria*;	VSN14	LC387504	0.0	1.4	58.5	66.0	89.9
Bacteria;*Proteobacteria*;*Gammaproteobacteria*;*Alteromonadales*;*Alteromonadaceae*;	VSN15	LC387505	0.0	0.0	26.6	4.0	+15.3
Bacteria;*Bacteroidetes*;*Bacteroidia*;*Flavobacteriales*;	VSN16	LC387506	0.0	1.4	10.6	5.3	11.5
Bacteria;*Bacteroidetes*;*Bacteroidia*;*Flavobacteriales*;*Crocinitomicaceae*;*Crocinitomix*;	VSN17	LC387507	2.9	1.4	62.1	17.2	18.6
Bacteria;*Bacteroidetes*;*Bacteroidia*;*Flavobacteriales*;*Flavobacteriaceae*;	VSN18	LC387508	0.0	1.4	0.0	14.5	10.5
Bacteria;*Planctomycetes*;*Planctomycetacia*;*Pirellulales*;*Pirellulaceae*;*Blastopirellula*;	VSN19	LC387509	1.4	2.8	23.0	34.3	13.6
Unclassified;	VSN20	LC387510	4.3	6.9	113.5	102.9	19.3
Unclassified;	VSN21	LC387511	0.0	1.4	21.3	9.2	22.0
Unclassified;	VSN22	LC387512	1.4	2.8	72.7	18.5	21.7
Unclassified;	VSN23	LC387513	0.0	1.4	0.0	17.2	12.4

a VSN indicates sequences obtained from NGS that increased in the $CHBr_3$-enriched treatment.

b The prefix of "+" indicates OTUs that were detected in the treatment bottles but were below the detection level in the control.

References.

1. **Fadrosh, D.W., B. Ma, P. Gajer, N. Sengamalay, S. Ott, R.M. Brotman, and J. Ravel.** 2014. An improved dual-indexing approach for multiplexed 16S rRNA gene sequencing on the Illumina MiSeq platform. Microbiome **2:**6.
2. **Herlemann, D.P.R., M. Labrenz, K. Jurgens, S. Bertilsson, J.J. Waniek, and A.F. Andersson.** 2011. Transitions in bacterial communities along the 2000 km salinity gradient of the Baltic Sea. ISME J. **5:**1571-1579.
3. **Kataoka, T., H. Yamaguchi, M. Sato, T. Watanabe, Y. Taniuchi, A. Kuwata, and M. Kawachi.** 2017. Seasonal and geographical distribution of near-surface small photosynthetic eukaryotes in the western North Pacific determined by pyrosequencing of 18S rDNA. FEMS Microbiol. Ecol. **93**.
4. **Ludwig, W., O. Strunk, R. Westram,** ***et al.*** 2004. ARB: a software environment for sequence data. Nucleic Acids Res. **32:**1363-1371.
5. **Muyzer, G., E.C. de Waal, and A.G. Uitterlinden.** 1993. Profiling of complex microbial populations by denaturing gradient gel electrophoresis analysis of polymerase chain reaction-amplified genes coding for 16S rRNA. Appl. Environ. Microbiol. **59:**695-700.
6. **Oksanen, J., R. Kindt, P. Legender, B. O'Hara, G.L. Simpson, M.H.H. Stevens, and H. Wagner.** 2008. vegan: Community Ecology Package, v1.13-1. http://vegan.r-forge.r-project.org/.
7. **Ooki, A., and Y. Yokouchi.** 2011. Dichloromethane in the Indian Ocean: Evidence for in-situ production in seawater. Mar. Chem. **124:**119-124.
8. **Pruesse, E., J. Peplies, and F.O. Glöckner.** 2012. SINA: Accurate high-throughput multiple sequence alignment of ribosomal RNA genes. Bioinformatics **28:**1823-1829.
9. **Quast, C., E. Pruesse, P. Yilmaz, J. Gerken, T. Schweer, P. Yarza, J. Peplies, and F.O. Glöckner.** 2013. The SILVA ribosomal RNA gene database project: improved data processing and web-based tools. Nucleic Acids Res. **41:**D590-D596.
10. **Rognes, T., T.s. Flouri, B. Nichols, C. Quince, and F.d.r. Mahé.** 2016. VSEARCH: a versatile open source tool for metagenomics. PeerJ **4:**e2584.
11. **Schmieder, R., Y.W. Lim, and R. Edwards.** 2012. Identification and removal of ribosomal RNA sequences from metatranscriptomes. Bioinformatics **28:**433-435.
12. **Suzuki, M.T., C.M. Preston, F.P. Chavez, and E.F. DeLong.** 2001. Quantitative mapping of bacterioplankton populations in seawater: field tests across an upwelling plume in Monterey Bay. Aquat. Microb. Ecol. **24:**117-127.
13. **Tanabe, A.S., and H. Toju.** 2013. Two new computational methods for universal DNA barcoding: A benchmark using barcode sequences of bacteria, archaea, animals, fungi, and land plants. PLoS One **8:**e76910.

Supplemental Fig. S1

Time evolution of $CHBr_3$ concentrations. Closed and open symbols indicate treatment and control, respectively. Error bars indicate variance in duplicate bottles.

Supplemental Fig. S2

Rarefaction curves of 97% similarity-based OTUs in each incubation on day 13. OTUs constructed by a single read from a single sample (singleton) were included in the left panel and removed in the right panel.

Supplemental Fig. S3

Relative read abundance of rank 3 in the SILVA taxonomic path that corresponds with class level taxa. Heatmap showing the relative read abundance/100,000 reads in each library. The sample name, which corresponds to that indicated in Fig. 2, indicates the two replicates of the control and treatments on day 13 of cultivation.

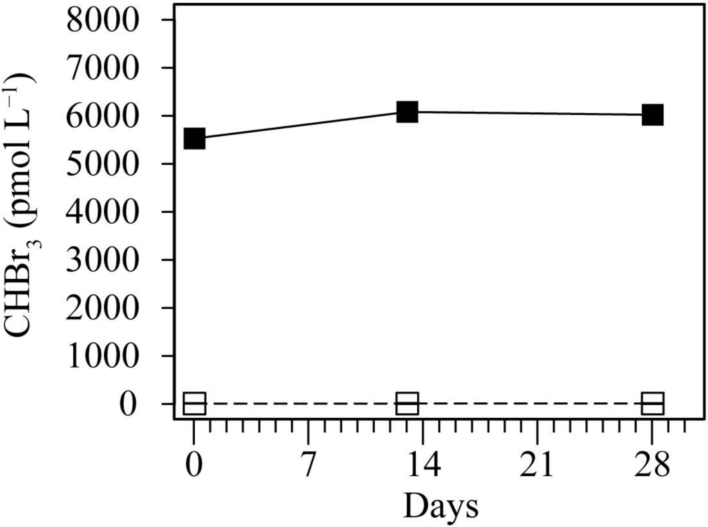

Kataoka et al. Fig. S1

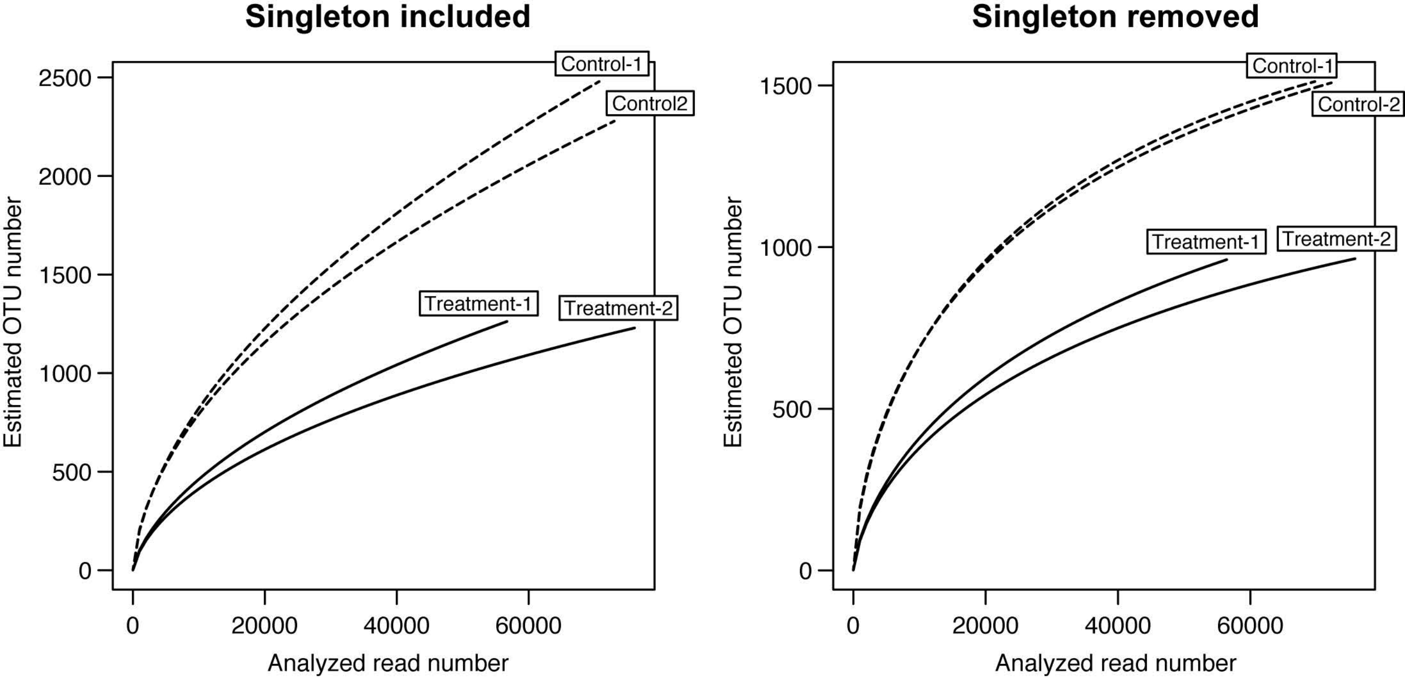

Kataoka et al. Fig. S2

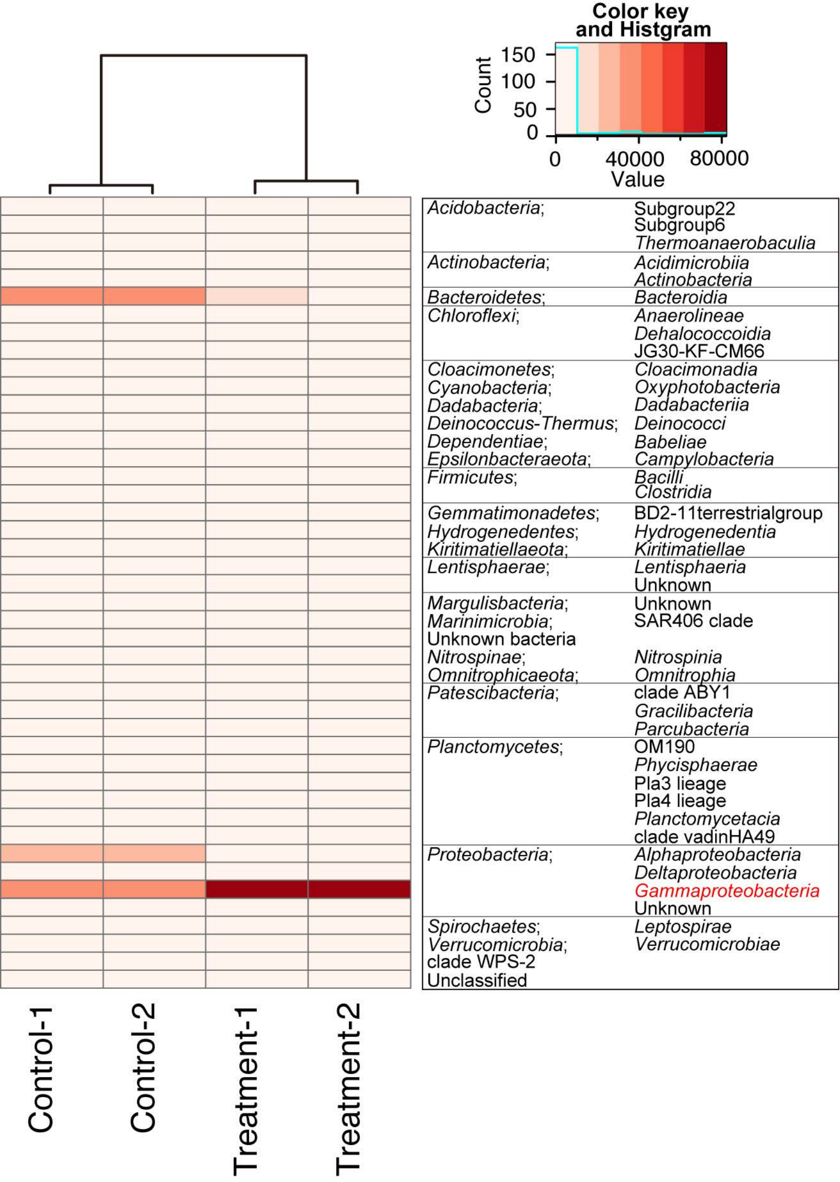

Kataoka et al. Fig. S3
